# Sensory Integration in Human Movement: A New Brain-Machine Interface Based on Gamma Band and Attention Level for Controlling a Lower-Limb Exoskeleton

**DOI:** 10.3389/fbioe.2020.00735

**Published:** 2020-09-03

**Authors:** Mario Ortiz, Laura Ferrero, Eduardo Iáñez, José M. Azorín, José L. Contreras-Vidal

**Affiliations:** ^1^Brain-Machine Interface Systems Lab, Miguel Hernández University of Elche, Elche, Spain; ^2^Laboratory for Non-invasive Brain Machine Interfaces, Department of Electrical and Computer Engineering, University of Houston, Houston, TX, United States

**Keywords:** brain-machine interface, EEG, gamma band, lower-limb exoskeleton, motor imagery, human movement, sensory integration, Stockwell Transform

## Abstract

Brain-machine interfaces (BMIs) can improve the control of assistance mobility devices making its use more intuitive and natural. In the case of an exoskeleton, they can also help rehabilitation therapies due to the reinforcement of neuro-plasticity through repetitive motor actions and cognitive engagement of the subject. Therefore, the cognitive implication of the user is a key aspect in BMI applications, and it is important to assure that the mental task correlates with the actual motor action. However, the process of walking is usually an autonomous mental task that requires a minimal conscious effort. Consequently, a brain-machine interface focused on the attention to gait could facilitate sensory integration in individuals with neurological impairment through the analysis of voluntary gait will and its repetitive use. This way the combined use of BMI+exoskeleton turns from assistance to restoration. This paper presents a new brain-machine interface based on the decoding of gamma band activity and attention level during motor imagery mental tasks. This work also shows a case study tested in able-bodied subjects prior to a future clinical study, demonstrating that a BMI based on gamma band and attention-level paradigm allows real-time closed-loop control of a Rex exoskeleton.

## 1. Introduction

Stroke, spinal cord injury (SCI), and limb loss are some of the most common causes of acquired motor disabilities in adults, being the restoration of motor function often incomplete. Normally, therapists try to recover some residual ability for movement when possible, acting over the distal physical level, trying to influence the neural system through mechanisms of neural plasticity (Ang and Guan, 2013). Traditional therapies focus on improving the functional ambulation for patients in the sub-acute phase, using overground training. This requires the design of preparatory exercises, the observation by a physical therapist and the direct manipulation of the limbs during gait over a regular surface, followed by supervised walking. Orthesis and prosthesis devices have been developed in the last years in order to assist people with motor limitations (Contreras-Vidal et al., 2016). The introduction of these robotic devices into rehabilitation therapies can further improve them (Bortole et al., 2015). Regarding the control, EMG-based interfaces can be used to control prosthesis (Villarejo Mayor et al., 2017), but a Brain-Machine Interface (BMI) offers a more suitable option to control a mechanical device, such as a speller or a wheelchair (Li et al., 2014), and exoskeletons or robotic orthesis (Do et al., 2013; Kilicarslan et al., 2013; López-Larraz et al., 2016; Liu et al., 2017). In addition, a BMI can improve neuroplasticity during rehabilitation therapies through the cognitive engagement of the subject (Cramer, 2008; Gharabaghi, 2016; Barrios et al., 2017), a fact that has been proved in clinical studies (Donati et al., 2016).

One of the most common paradigms used for BMIs to decode the brain activity is motor imagery (MI). It has been demonstrated that the mental task of imaging a movement produces similar brain patterns to the actual motion (Stippich et al., 2002; Bakker et al., 2007; Batula et al., 2017). Feature extraction of MI is usually based on the frequency analysis of the subject's electroencephalographic signals (EEG) in alpha (8 − 12 Hz) and beta bands (12 − 32 Hz) (Pfurtscheller et al., 2006), or delta bands (0.1 − 4 Hz) (Bradberry et al., 2010; Presacco et al., 2011). However, there are not many studies that focus on gamma bands (32 − 100 Hz). Recently, the gamma band has been related to gait attention (Costa et al., 2016; Costa-García et al., 2019). However, the actual action of walking does not demand high attention from the individual, as it is usually involving a subconscious mental task. Besides, the subject can be affected by external sensory distractions that can reduce the level of cognitive engagement associated with the MI task. For this reason, it is important to assess the attention level that the subject keeps during the mental task of controlling the robotic device. This way, it can be assured that the cognitive engagement of the subject is high during the therapy, and that the control outputs are accurate and associated with the mental process of rehabilitation. This allows to turn assistive BMIs into restorative BMIs (Gharabaghi, 2016).

The present research combines two different paradigms in order to propose a new BMI for controlling a lower-limb exoskeleton. First, a new BMI based on MI for gamma band is presented. The current work expands the initial research developed in Ortiz et al. (2019), studying the real-time feasibility of the new MI paradigm in an opened-loop and closed-loop control scenario. Second, the BMI proposed in Costa-García et al. (2019) is adapted to the current research in order to evaluate the attention to gait based on a dual task paradigm. The attention level provides this way, a measurement of the cognitive engagement of the subject during the use of an exoskeleton, fact that has not been studied previously in literature. This information could be provided during rehabilitation therapies to the subject and clinical staff to assess the degree of engagement during the MI mental task. Finally, the viability of the combination of the attention level to gait as a modifier for the initial MI paradigm is studied. The objective is to see if the combination of both paradigms allows to operate the exoskeleton with a higher accuracy. This new approach has been tested with several able-bodied volunteers, as a preliminary study before its employment with patients in a second stage of the research. The results show that the proposed BMI can be used for real-time closed-loop operation of a Rex exoskeleton.

## 2. Materials and Methods

This section describes the experimental setup, the equipment used, the data processing methods and the indices used for assessment.

### 2.1. Equipment

**Figure 1:**
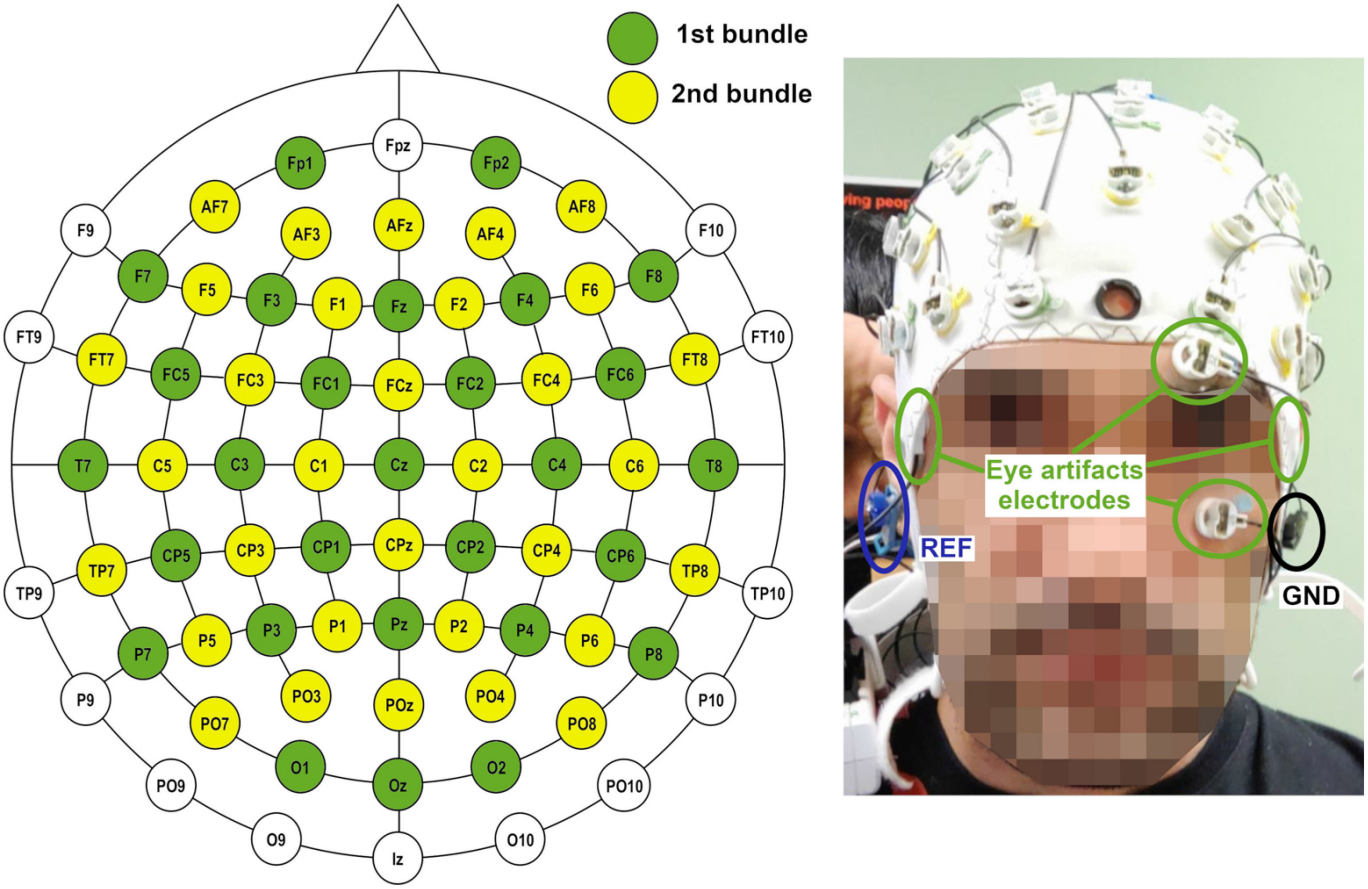
Electrode configuration for the experiments. Sixty of the electrodes were used for EEG recording. Four of the electrodes of the first bundle were used for assessing eye artifacts. Ground and reference were positioned on left and right ear, respectively.

Data acquisition was accomplished by two non-invasive bundles of 32 wet scalp electrodes over an easyCap unit (Brain Products GmbH, Germany). The cap followed the 10–10 distribution of the international system. Four of the electrodes of the first bundle (see Figure 1) were placed around the eyes in a bipolar setup to assess the contribution of blinking to artifacts. Reference and ground electrodes were positioned on both ears. Data were transferred by wireless communication using a Move transmitter (Brain Products GmbH, Germany) for their posterior amplification by two brainAmp units (Brain Products GmbH, Germany) and their processing and recording in a laptop.

The exoskeleton used was the Rex (Rex Bionics, New Zealand). The exoskeleton was controlled by wireless communication. The feedback information of the current status of the Rex was acquired by the computer through a wire serial port communication with a custom developed software. Rex exoskeleton has several characteristics which make it different from other lower-limb exoskeletons. First, it is a self-standing exoskeleton that does not require any crutches and that allows a full standing walking without any vertical inclination. In addition, its walking pattern is very peculiar and far from the anthropomorphic usual gait. The choice of this exoskeleton was made based on the movement limitations it provides. In a Rex exoskeleton, the limbs of the subject are tightly attached to the robotic prosthesis by several straps, avoiding any lower limb movement. This way, the subject can only move their legs when the exoskeleton does, avoiding any lower-limb movement not commanded by the BMI.

### 2.2. Experimental Setup

#### 2.2.1. Subjects

Four able-bodied subjects (S1–S4) took part in the experiments. The subjects did not report any known disease and participated voluntarily in the research, giving written and informed consent. All the procedures were approved by the Institutional Review Board of the University of Houston, TX (USA). The research included two different experiments. S1 and S2 participated in the initial opened-loop experiments, which were used to set up the initial algorithms of both BMIs (only MI and MI+attention). Additionally, subjects S2–S4 participated in the sessions which were designed with the objective to test the initial results in a closed-loop control scenario. S1 could not participate in the closed-loop experiments due to malfunctioning of the electrodes.

#### 2.2.2. Subject Preparation

Preparation of the subject included two different steps. First the limb length of the exoskeleton was adjusted to the subject. After that, the electrodes were gelled to a value lower than 30*kΩ*. Electrode's impedance was checked before starting the trials and after finishing to be sure no electrodes were marginally over the 30*kΩ* value. Full process for both tasks could take around an hour. Before starting data collection, a medical mesh was positioned over the cap to avoid any wire movement and mitigate motion artifacts. Before starting, several runs of walking by manual control were accomplished in order to get the subject used to the Rex movement.

#### 2.2.3. Protocols

**Figure 2:**
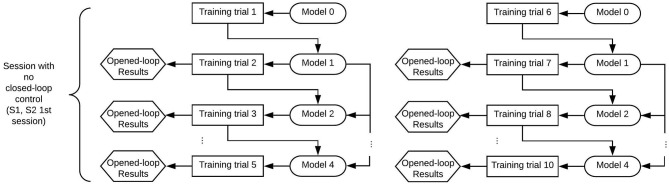
Structure of a session with only opened-loop control per paradigm of control. The trials were registered and computed for a determined paradigm of control (MI or MI+att) in groups of five trials. Each session consisted of 10 training trials, which were recalculated for the other paradigm of control in a pseudo-analysis (1 − 5 and 6 − 10). The model used for testing each trial in opened-loop included the previous n-1 trials up to a maximum of four trials.

**Figure 3:**
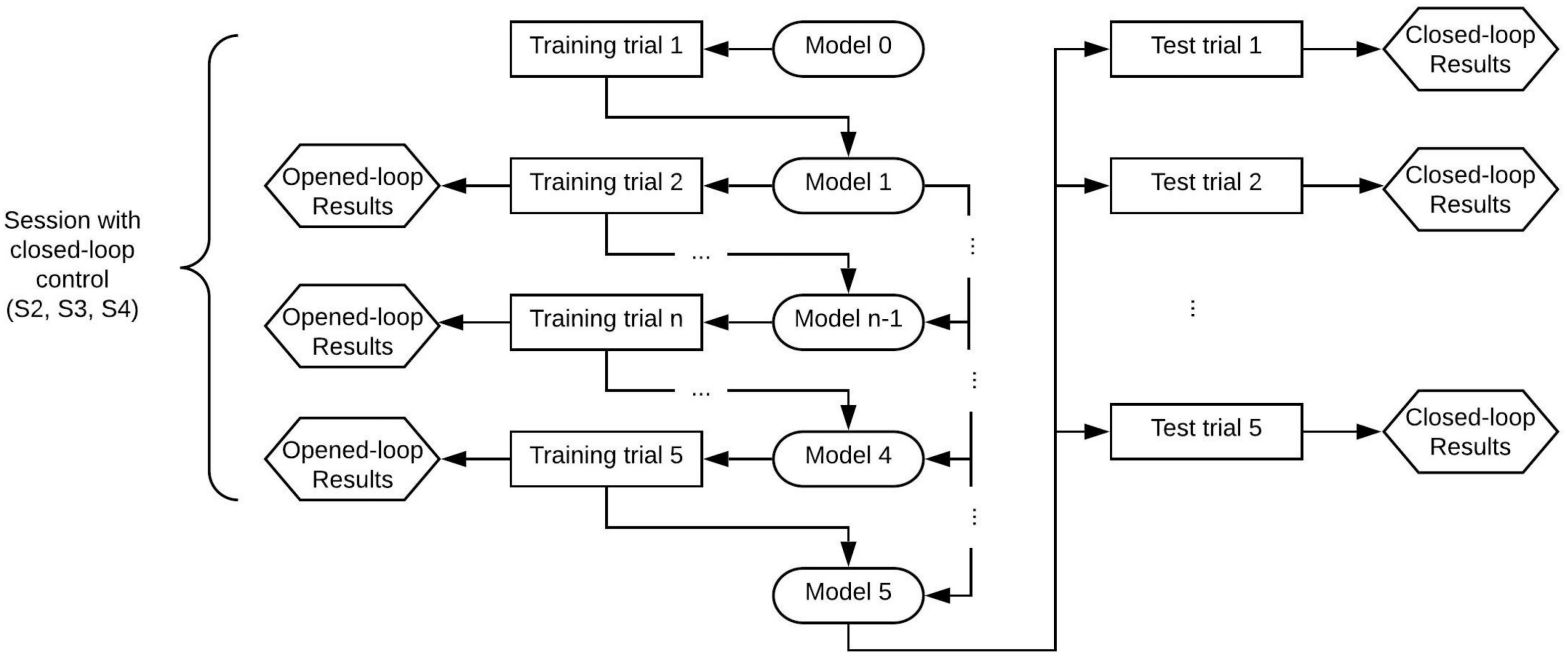
Structure of a session with closed-loop control per paradigm of control. The subject performed the whole diagram once per each paradigm of control (MI and MI+att). Each paradigm consisted of five training and five tests trials, so each session consisted of 20 trials. The test trials were tested with the specific trained model.

Figures 2, 3 show the structure of both kind of sessions (only opened-loop control and with closed-loop control). First sessions of the research included only training trials that were controlled in opened-loop. Once the paradigms were set-up based on the first sessions data, experimental sessions included test trials which were controlled in closed-loop. Following paragraphs detail the characteristic of both kind of trials.

##### 2.2.3.1. Training trials

Training trials were the ones used for creating the classifier model of each paradigm control (MI or MI+attention). As the developed tool always works in real time, first training trial needs a generic model 0 (randomized data) in order to be processed. The output results of the first training trial were not considered for this reason, as the trial was classified with a model which contains data that was not related to the subject. Subsequent training trials were tested in opened-loop control with the model of the previous n-1 trials of the subject. As real-time analysis can be done for only a specific algorithm of control (MI or MI+attention), trials were run with an specific kind of model paradigm. However, a pseudo-online analysis for the other control paradigm was run to compare the performance of both paradigms, as it will be seen in results section.

**Figure 4:**
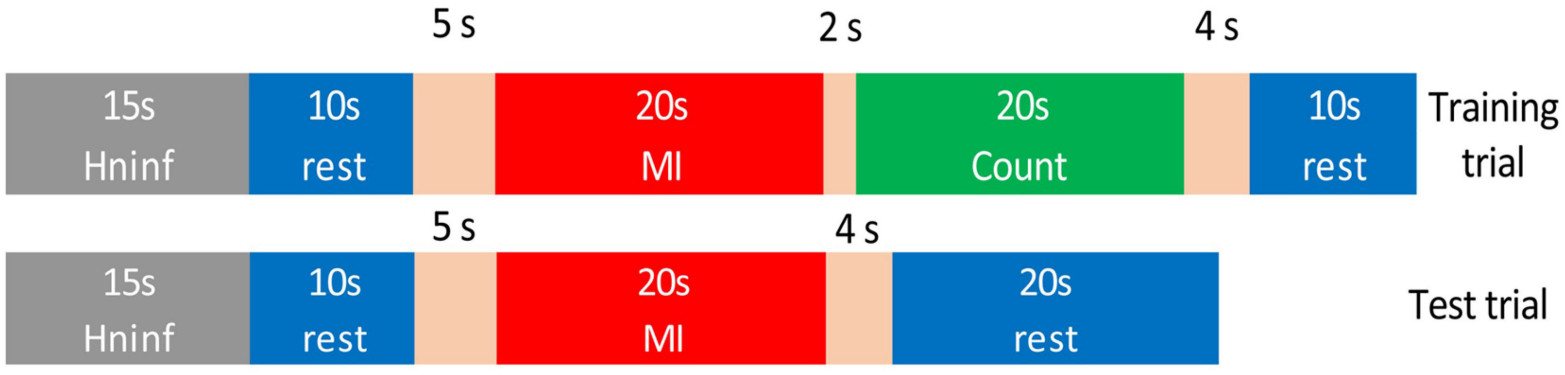
Times of the mental events during the experimental trials. Both trials included a previous time of 15 *s* followed by a 10 *s* rest period. In the case of training trials, this period was followed by walk/mathematical count/stop events. Test trials for testing did not include the mathematical count event and had an extended final stop event to allow the Rex to stop. As first and last steps of the exoskeleton had a variable time, extra windows of time of 5 *s* for the start and 4 *s* for the stop were considered.

**Figure 5:**
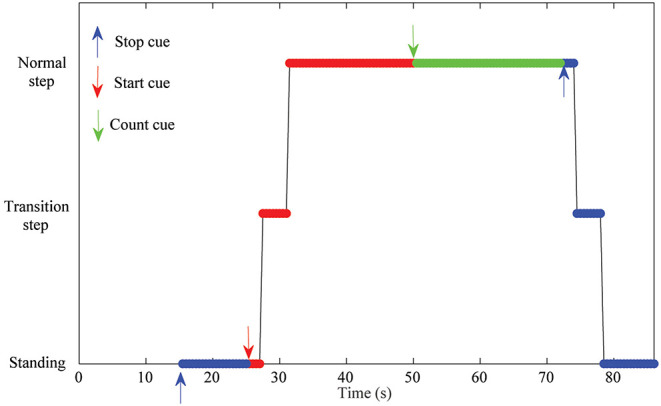
Experimental protocol for the training trials. Each event started/finished with an acoustic cue (red/blue/green arrows for start, stop, counting events). The figure also shows the Rex status during the trial (Standing/transition step to walk or stop/Normal walking). First 15 *s* were not considered for analysis and were used to allow the artifact removal algorithm to converge. As it can be seen there is a hardware lag in the status of the Rex since a start or stop cue is issued and the Rex changes its status.

The protocol of a training trial included three different mental tasks (see Figure 4). First, 15 *s* were not used for classification as they were needed for the convergence of the *H*^∞^ eye blinking artifact removal algorithm (Kilicarslan et al., 2016). In addition, these seconds helped the subject to feel relaxed before the start of the trial. After that, an acoustic cue marked the start of a rest/standing event, which ended by another acoustic cue for starting the MI event and the Rex activation. After at least 20 *s* of normal exoskeleton walking, a new acoustic cue indicated the start of a reverse mental count. This mathematical task substituted the original mathematical operations used in Costa-García et al. (2019) for the assessment of the low attention to gait, as it was difficult to attach a tablet to the Rex exoskeleton without disturbing the subject. The mental operation consisted of an accumulative counting of 1, 000 ± 7. The ± was changed randomly between trials to avoid the repetition of the numbers and any memorization of the operations by the subject. The counting event was used as a distracting mental task to assure that the focus of the subject was not on the gait during the Rex walking. This counting also worked as a control class to detect if the output differences in the MI class were related to motion artifacts. As the Rex is moving in the same way during MI and counting periods, the output differences were just related to the mental processes. In addition, to take into account the time that the exoskeleton needs to perform the transition step to start or to stop, additional windows of time of 5 *s* for the start (2 *s* for the cue influence +3 *s* for the transition step) and 4 *s* for the stop were considered. Status of the Rex can be seen in Figure 5 operating in an opened-loop control. As it can be seen in the image, there is some inherent lag since the real command is issued, which in training trials is coincidental with difference between the acoustic cue (start or stop) and the moment the Rex initiates the transition step or achieves the reference status (normal walking or standing position).

##### 2.2.3.2. Test trials

Test trials were run in real-time closed-loop control in order to assess the BMI behavior. The model that used the test trials consisted of the previous five training trials acquired.

The trials used for testing the BMIs were similar to the training ones, but without the count event, see lower part of Figure 4. In order to give enough time for the Rex to stop, the final rest event was expanded to 20 *s*, as the lag introduced by the Rex between the issue of a stop command and an actual stop can last over 7 *s* depending on the position of the mechanical limbs. This is because final standing position must leave both limbs in parallel, requiring sometimes to fulfill a full last step plus the transition step before stopping. The command to start or to stop the Rex was issued only when a decision command output was created by the BMI based on the output of the classifier.

### 2.3. Signal Processing

**Figure 6:**
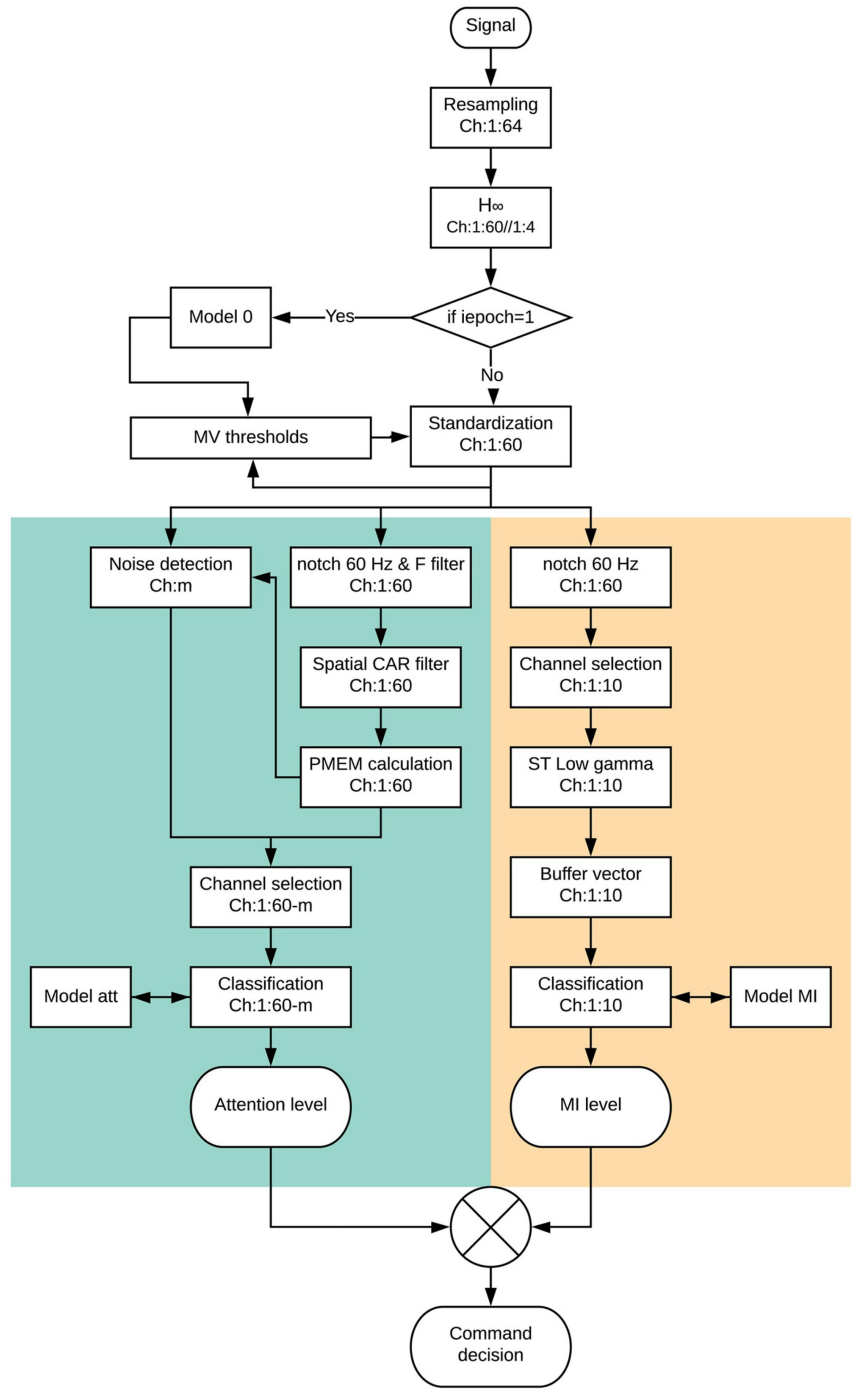
Scheme of the full processing of an epoch since it is acquired and an output command decision is interpreted. The processing is carried out in the same way for the pseudo and the online analysis in real time. The only difference is that during pseudo-online analysis the command decision is not sent to the exoskeleton. Attention paradigm part is in green while MI paradigm is in beige.

The whole signal processing scheme can be seen in Figure 6. The following paragraphs will describe it for the different processing paradigms.

#### 2.3.1. Initial Pre-processing

One of the most difficult problems a BMI based on non-invasive EEG must confront is the presence of artifacts which could spoil the information contained in the EEG signal. This is especially difficult in the case of real-time algorithms, as no offline analysis mitigation is possible. The movement of the Rex is slow enough to not have important motion artifacts. Nevertheless, the electrode's wires were carefully attached using plastic clamps and a medical mesh was placed over the cap in order to avoid any fluctuation of the wires. In addition, subjects were advised to avoid any swallowing or chewing during the experiment. In order to mitigate the artifacts associated with eye blinking, *H*^∞^ (Kilicarslan et al., 2016) algorithm was applied (see Figure 1 for detail of the electrodes used for *H*^∞^). As the algorithm needed to work in a real-time scenario, the sampling frequency of the original data was resampled from 1 *kHz* to 200 *Hz*, applying each sample for a variable state function for an anti-aliasing low-pass filter. The filter was designed to maintain the Shannon sampling theorem requirements, with cut-off frequency equal to the new Nyquist frequency (Vaidyanathan, 1993). The 200 *Hz* frequency was chosen as a compromise between frequency resolution (allowing the extraction of γ band) and the processing speed of an epoch of 1 *s* length. This value was based on the preliminary analysis of former Rex data by the lab and the first of the opened-loop sessions. Time processing was an important issue as each epoch shifted every 0.5 *s*. Therefore, the whole processing time since an epoch was collected and a command decision was taken (see Figure 6) should be below 0.5 *s* for all the epochs in a trial. This was accomplished for the pseudo-online and online analyses requiring and optimization of the code not usually needed in an offline analysis.

After the signal was resampled and free of ocular artifacts, a standardization process was accomplished. This step is important to give the same weight to different electrodes for the MI and attention paradigms and to avoid that the changes in the EEG voltages of the subject between training and test trials can affect the classification. The standardization process was similar to the one presented in Costa et al. (2016) using the maximum visual threshold (MV). For each electrode *i*, it is computed based on its voltage *V*, and updated for each epoch *m* = 1 : *N* of the trial, with a length of *L* samples as:

(1) $$\begin{array}{r} MV^{i}=\frac{1}{N}\overset{N}{\underset{m=1}{\sum }}max\left[abs\left(V_{\left(m-1\right)\cdot L+1:m\cdot L}^{i}\right)\right] \end{array}$$

Being the standardized voltage of the electrode *i* per each sample at time *t*:

(2) $$\begin{array}{r} SV^{i}\left(t\right)=\frac{V^{i}\left(t\right)}{\frac{1}{60}\sum_{j=1}^{60}MV^{j}} \end{array}$$

For the first epoch of the first training trial, the BMI takes the MV thresholds of the generic model 0 file, based on the data of a former subject. This information is updated for each epoch, converging to a stable value after several seconds, so it does not affect the epochs in the events under analysis. Following trials use the updated thresholds of previous trials, so model 0 is just used for the initial seconds of the first training trial as a way to accelerate the MV convergence.

#### 2.3.2. Attention Level Paradigm

The attention level BMI is based on the previous research published in Costa-García et al. (2019). However, it is particularized for the 60 EEG channels of the proposed setup instead of the original 31 channels, and the mental tasks of this research, as it is not possible for the Rex to follow ground stamps.

The first step is to detect the presence of any residual noise in the signal. An epoch of electrode *i* is considered as noisy if the instant MV threshold is over 150 μ*V*, its instant kurtosis is over 15 μ*V*, and its spectral power obtained by the maximum entropy method is not over 14 μ*V*^2^.

Regarding the data processing, first a notch filter at 60 Hz and a fourth order Butterworth band-pass filter (5−90 Hz) is applied to the epoch, followed by a spatial Common Average Reference filter (McFarland et al., 1997). After that, the power spectral density of the channel is computed by the maximum entropy method (Rainford and Daniell, 1994) for the bands between 30–55 and 65–90 Hz. Bands were changed from the original research due to the different line frequency in the USA.

In order to consider an epoch as valid for attention level assessment, at least 21 channels must be not noisy. If this is not the case, the attention level is considered as the one of the previous epoch (please check Costa-García et al., 2019 for further information of the method). This means that the feature data vector can range between 21 and 60 data. Nevertheless, due to the extended number of channels in this research, and the preliminary artifact filters, this was not needed in practice, as the noise content was below the original research.

For the classification, three different classes were considered: rest, count and walk. Rest class contains the epochs of the standing rest periods of the Rex (standing blue parts in Figure 5, about 20 *s*); count data consists of the 20 *s* of walking mathematical operations (green part in Figure 5 neglecting the 2 *s* after the count cue); and walk class is based on the normal step walking periods (upper red part in Figure 5, about 20 *s*). Due to the uncertain time needed to do the transition steps, rest and walk periods can have slightly different number of epochs. However, the difference is not high enough to unbalance the classes.

The classifier uses a Linear Discriminant Analysis (LDA) algorithm, which is a generalization of Fishers Discriminant classifier (Izenman, 2013). For the opened-loop analysis, the model is created using the data-set of the previous training trials. This means that trial *i* is classified with the information of training trials 1 : *i* − 1. In the case of the closed-loop testing, the model is created with the five associated training trials (see Figures 2, 3). The first training trial needs a generalized model 0 to be processed as there is not previous model data and the tool works always in real-time for all the registers. This output can not be used for assessment as it is not relevant. This is the reason why opened-loop analysis shows only the information of training trials 2 − 5.

**Figure 7:**
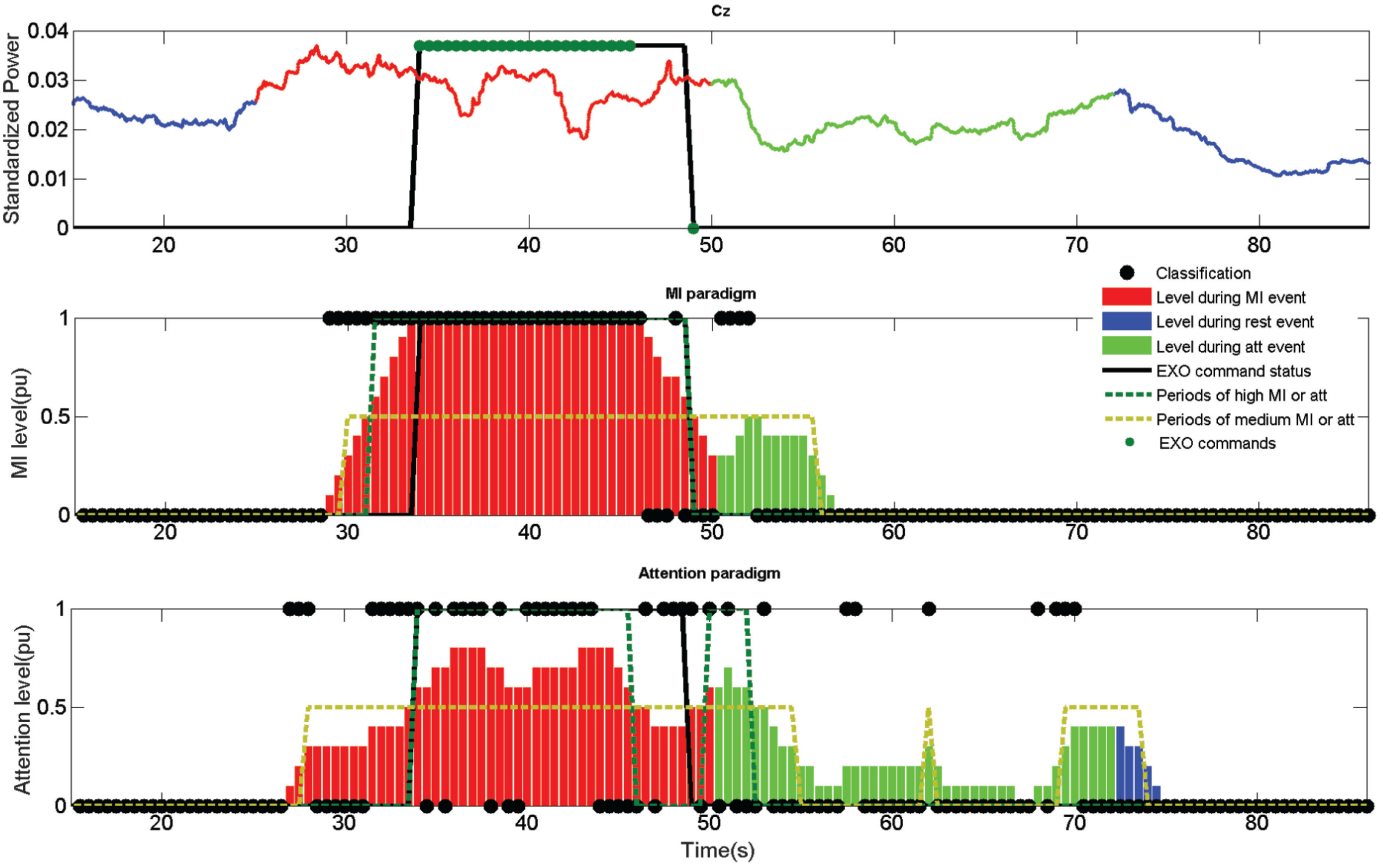
Output information of the fifth opened-loop training trial of subject S1. Up image shows the output of the MI paradigm features for electrode Cz. Center image shows the MI classifier output. Down image shows the attention classifier output. Mental tasks are color coded (Blue for rest, red for walk and green for count). MI and attention levels are shown for each paradigm in bars ranged from 0 to 1. A high level is considered when the bars are above 0.5 (green dotted) and a medium level when it is above 0.25 (golden dotted). Classifier outputs are shown as •, and exoskeleton commands as •. Rex command status is represented as a thicked black line, which would represent the status of the exoskeleton in the case the commands could be issued instantaneously without any hardware lag. The exoskeleton was commanded for this example using the MI+att paradigm based on the periods of high and medium MI and attention.

After each epoch is classified, a weight is assigned depending on the output label. If the output corresponds to a rest or count label, an attention value of 0 is assigned and if it is a walk a 1. This value is then averaged for the last 10 epochs. This means that for obtaining a maximum attention value of 1, 10 consecutive epochs must be classified (5 *s* due to the shifting). Considering the data acquisition lag (+0.5 *s*) means that at least 5.5 *s* are needed to achieve a perfect attention level. This is important to understand that certain lag is inherent to the assessment method. This way, a medium level attention is considered when it is over 0.25 and a high level over 0.5 (around 3 *s* of consecutive walk detection). An example of the attention level can be seen in Figure 7 as the bars of the attention paradigm image (down). Each of the classifications can be seen in the image as a •.

#### 2.3.3. Motor Imagery Paradigm

The MI BMI is based on the preliminary study developed in Ortiz et al. (2019). In that research, a BMI based on γ band was presented and the conclusion extracted was that γ band could be used for commanding an exoskeleton with a low false positive ratio. However, the study was limited to one subject and tested in an offline scenario. In the present research some changes have been done to the former BMI to allow its use in real time and in coordination with the attention level BMI for commanding the Rex exoskeleton in closed-loop control.

As it can be seen in Figure 5, data is first notch filtered at 60 Hz. The rest of the processing is applied to the central electrodes associated with MI tasks: Fz, FC1, FCz, FC2, C1, Cz, C2, CP1, CPz, and CP2.

Regarding the feature extraction, this is done using Stockwell Transform (ST) for each epoch (Stockwell et al., 1996). Although ST is applied to the whole epoch (1 *s*), in order to avoid border effects the information considered is extracted from 0.25 to 0.75 s of each epoch. This means, that each epoch overlaps information for a quarter of second, as epochs are shifted at a 0.5 *s* pace. Once ST is computed, the instantaneous power of the voices from 30 to 55 Hz lower γ band is added. This changes from the original research that used the maximum peaks of the low and high γ bands. Preliminary studies using the S1, and S2 training data revealed that high γ band did not produce a significant improvement of accuracy, while its no consideration kept the processing times below the shifting time. Besides, the addition of power, instead of the computation of the maximum peak, produced slightly better results without affecting the computation times.

The buffer for smoothing the output was extended to 4 *s* in this research (this contains data since 4.5 *s* due to +0.5 *s* needed for acquisition). This is a compromise between 3 *s* needed for a medium attention level and the 5.5 *s* for a high attention level. An example for the smoothed value of electrode Cz can be seen as standardized power in the upper image of Figure 7.

This smoothed value is averaged afterwards for each epoch to obtain the associated feature of each electrode. This is another difference in comparison with the former research, as the calculation of the features is done by each individual electrode and epoch and its value is not averaged for the 10 electrodes (Ortiz et al., 2019). This allows to use the 10 features per epoch as a vector data for the LDA classifier.

Regarding the classifier, only two classes are considered, walk and rest, instead of the three classes of the attention model. Similarly to the attention BMI, depending on the output label of the classifier, a 0 is assigned to each epoch for a rest detection and a 1 for walk. This way, the MI level is computed in an analog way to the attention paradigm, see MI paradigm (center) image of Figure 7. In the same way than the attention paradigm, the MI level is shown as bars and the classifier output as a •.

#### 2.3.4. Command Decision

Once the MI and attention levels are assessed, they are used to create a decision for the output command of the BMI. In the case of the opened-loop trials, the information is recorded for its evaluation, while in the case of closed-loop trials it is sent to the exoskeleton providing feedback to the subject. Two different command rules were tested depending on the paradigms used: MI and MI+att.

**Figure 8:**
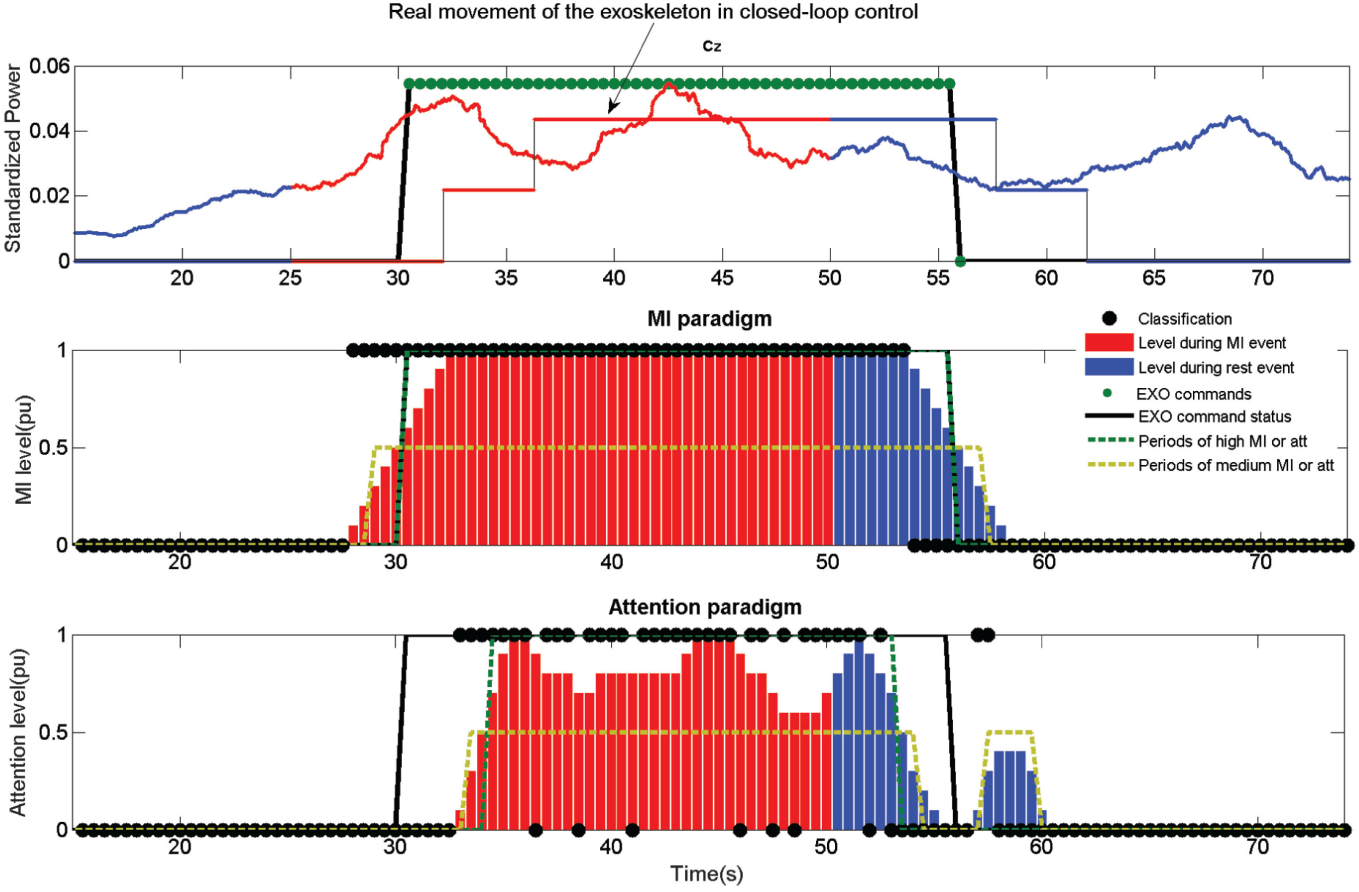
Output information of the first closed-loop test trial of subject S2. Information is presented in a similar way to Figure 7. In the upper image it is also included the actual movement status of the exoskeleton caused by the EXO commands. As it can be seen, there is a certain lag between the EXO command status and the actual movement due to the hardware (difference between the thicked black line and the thin black line in the upper image). The exoskeleton was commanded for this example using just the MI paradigm, neglecting the information provided by the attention paradigm. The combination of both paradigms would have result in a shorter movement following the rules in subsection 2.3.4 issuing the activation command in the 33 *s* approximately instead of the 30.5 *s*.

First control method (MI) only uses the information of the MI levels, creating an output command when its value is over 0.5. To simulate the time needed for the exoskeleton to finish a step, it is not possible to send two different commands in a timelapse of 3 *s*. This does not affect the behavior of the control in a closed-loop scenario, as the hardware cannot process opposite commands during a step. However, it provides a more realistic output of the commands and indices for opened-loop trials. An example of a closed-loop control using just the MI control can be see in Figure 8.

Second control method (MI+att) combines both paradigms to make a command decision. The requisite of no-different commands within a 3 *s* period is also present. The rules are more complex and can be summed as:

- If no command was issued since 3 *s*.
  – If MI>0.5 and att> 0.5, walk (high MI and att).
  – If MI <0.5 and att< 0.5, stop (medium or less MI and att, can be accounted while in standing position).
  – if EXO command = 1 and MI< 0.5 and att< 0.25, stop (during walking, medium or low MI with low att).
- else
  – If EXO command = 1 and MI_*epoc*_*h*__*i*__ > 0.25 and MI_*epoc*_*h*__*i*−1__ > 0.25 or att> 0.25, reinforcing walk (during walking, at least medium MI for current and previous epoch or medium att for current one).

Reinforcing commands are needed to assure that the exoskeleton goes on walking, as the absence of a walking command makes the Rex to stop.

### 2.4. Indices for Assessment the BMI Performance

One of the most important aspects to evaluate the performance of a BMI is to correctly define the indices considered for its evaluation. Literature is not always precise in the definition of them, which can cause difficulties to compare the results. This subsection tries to overcome this difficulty clearly defining all the indices that are going to be used for the BMI assessment in a quantitative and qualitative way.

#### 2.4.1. True Positive Ratio (TPR)

It indicates the percentage of walking events that are executed during a walking event. As the trials have only a walking event, this value can be only 0% or 100% per trial, indicating the average value the percentage of trials with true activations. The qualitative scale would be: poor < 50%, average ≥ 50%, good ≥ 65%, very good ≥ 75%, and excellent ≥ 85%.

#### 2.4.2. Accuracy (Acc)

It indicates the number of correct commands issued with respect to the total number of commands. A correct command is when a walk or stop command is computed in a walk or stop event, respectively. The qualitative scale is the same of TPR.

#### 2.4.3. False Positives (FP) and False Positives per Minute (FP/min)

This is one of the most important indices, as it quantifies the number of walking commands issued during rest or count periods. One of the objectives of the research is to kept this number as low as possible, even if it limits the accuracy of the BMI. For the real-time control of an exoskeleton, it is an important problem if the exoskeleton is activated when it is not desired, as it could be frustrating for the patient during therapies or make the control unusable for assistance. This index is usually computed per minute, which is also included for comparison of the different performances. The qualitative scale for the FP is: poor >2, average ≤ 2, good ≤ 1.5, very good ≤ 1, and excellent ≤ 0.5.

#### 2.4.4. Percentage per Epoch and Paradigm

**Figure 9:**
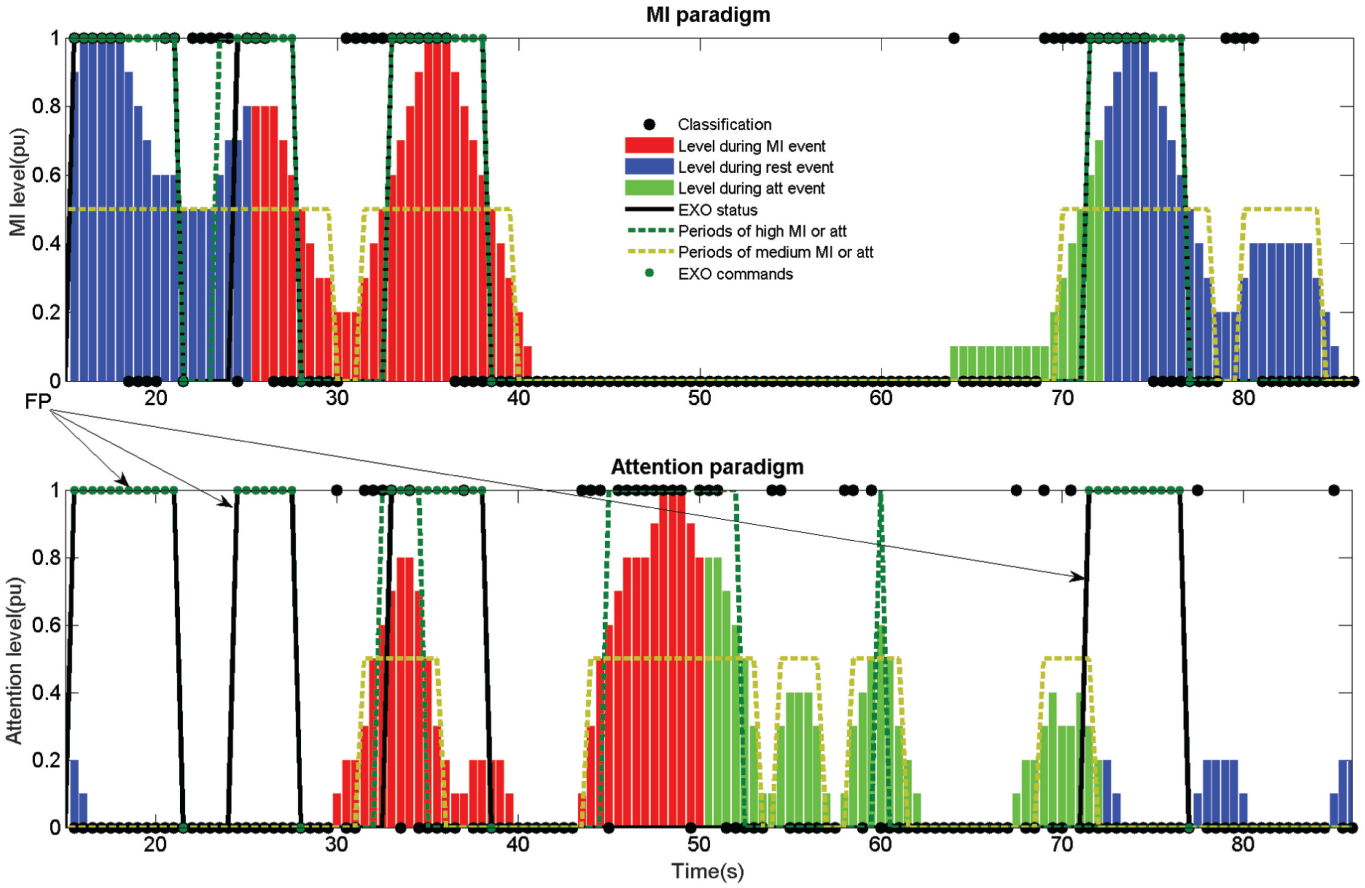
Example of an erroneous classification in an opened-loop trial. The image corresponds to the training trial 5 of subject S4. The poor %MI classification (52.8%) produced three FP and an Acc of 37.5%. If the attention paradigm had been considered, the three FP marked by the arrows would not have been computed. However, the exoskeleton would have been activated for a shorter period of time (between 33 and 38 s), stopping before the count event starts. This would have had a 50% Acc as the stop would have been commanded before the MI period ended. Compare Table 1 results for both paradigms of training trial 5 of S4.

There are three different indices: %MI, %att, and %Command. The first two indicate the accuracy of each classifier paradigm, as the number of correct detections divided by the number of total detections. Detections can be seen in Figures 7–9 as •. A correct detection value would be 1 for walk events and 0 for the other events. The third one is based on the percentage of epochs with correct EXO command status. It is important to notice that due to the way the algorithms work (averaging previous epochs and outputs in the case of the MI and attention levels), it is not possible to have a 100% accuracy as it would need at least several seconds of perfect features to compute a change of event. The qualitative scale would be: poor < 50%, average ≥ 50%, good ≥ 60%, very good ≥ 70%, and excellent ≥ 80%.

#### 2.4.5. Lags in Command

It can be computed for the commands (opened-loop) and for the exoskeleton (closed-loop). As the second one is hardware dependent, it does not provide any information of the BMI performance. They are a quantitative value of the time needed to change the status since a cue is established. For instance, the time needed to walk or to send the walk command since the walking acoustic cue is issued. There is not a qualitative scale, but a good value would be <10 s for the EXO command having in consideration the lag of the algorithms and the exoskeleton.

**Table 1:**
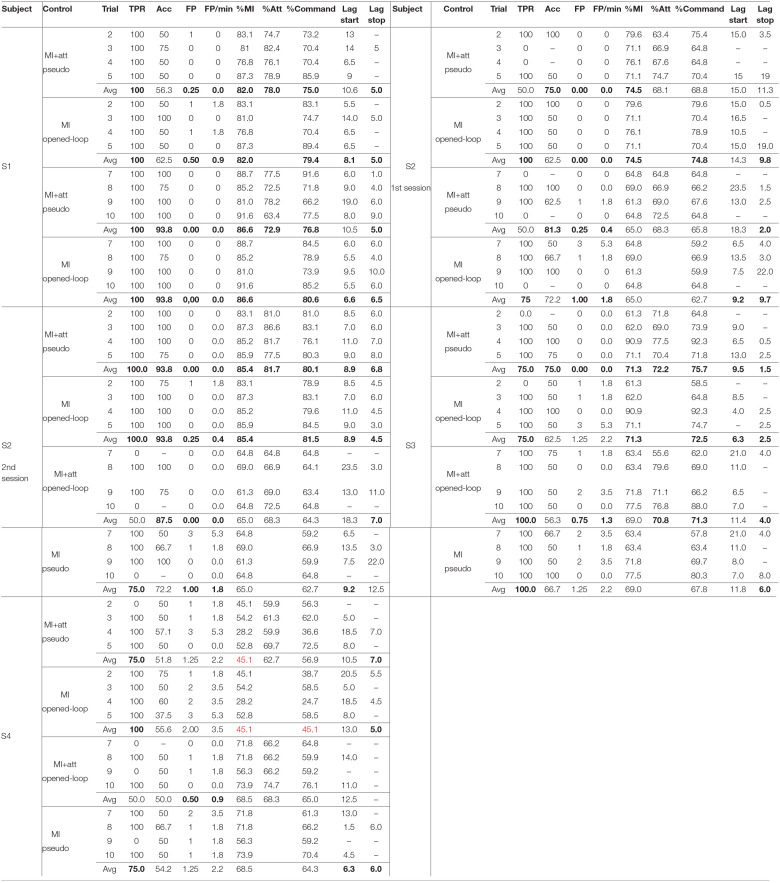
Results of the analysis of the training trials.

*The trials were also tested in a pseudo-online analysis for the type of control not used during the opened-loop session. Averaged very good results using the qualitative scale are in bold, while poor in red*.

## 3. Results

As previously stated, all the trials (training and test) were processed in a real-time (online) scenario. Training trials were executed in opened-loop control while test trials were executed in closed-loop control. Each trial was performed following a determined method of control (MI or MI+att). In the case of closed-loop control tests, the method of control corresponds to the one used when registered. However, in the case of training trials, they were simulated again using a pseudo-online analysis. For this reason, training trials show the results of both methods of control and test trials only the method that was actually executed in real time.

### 3.1. Opened-Loop Results

Table 1 shows the results obtained for the training trials. When there was not an activation of the exoskeleton, there is a “-” under Acc index. The results by subject and algorithm of control can be seen in Tables 2, 3 based on the section 2.4 indices.

**Table 2:** Results of the training trials averaged per method of control.

**Control**	**TPR**	**Acc**	**FP**	**FP/min**	**%MI**	**%Att**	**%Command**	**Lag start**	**Lag Stop**
MI+Att	**75.0** **±** **43.9**	70.9 ± 34.8	**0.30** ± **0.65**	**0.49** ± **1.13**	**71.2** ± **13.3**	**71.1** ± **7.0**	**70.0** ± **10.3**	11.8 ± 6.8	**5.6** ± **4.1**
MI	**90.0** **±** **30.4**	69.5 ± 26.8	**0.85** ± **1.00**	**1.50** ± **1.77**	**71.2** ± **13.3**		69.1 ± 13.2	**9.6** ± **5.5**	**6.8** ± **5.6**

*Averaged very good results using the qualitative scale are in bold. No poor results were achieved for the averaged indices per method of control*.

**Table 3:** Results of the training trials averaged per subject.

**Subject**	**TPR**	**Acc**	**Fp**	**Fp/min**	**%Mi**	**%Att**	**%Command**	**Lag start**	**Lag stop**
S1	**100.0** **±** **0.0**	**76.6** **±** **23.2**	**0.19** **±** **0.40**	**0.22** **±** **0.60**	**84.3** **±** **4.7**	**75.4** **±** **5.7**	**77.9** **±** **7.7**	**9.0** **±** **4.0**	**5.6** **±** **3.4**
S2	**75.0** **±** **44.0**	**80.0** **±** **21.8**	**0.31** **±** **0.78**	**0.55** **±** **1.38**	**72.4** **±** **9.0**	**71.6** **±** **6.9**	**70.1** **±** **8.0**	11.9 ± 4.8	**7.6** **±** **6.9**
S3	**87.5** **±** **34.2**	**64.4** **±** **20.5**	**0.81** **±** **0.98**	**1.43** **±** **1.73**	**70.2** **±** **9.8**	**71.5** **±** **7.4**	**71.8** **±** **11.2**	10.3 ± 5.3	**3.4** **±** **2.3**
S4	**75.0** **±** **44.7**	53.1 ± 8.7	**1.25** **±** **0.93**	**2.21** **±** **1.64**	56.8 ± 15.2	65.5 ± 5.1	57.8 ± 13.6	10.6 ± 6.3	**5.8** **±** **1.0**

*No poor results were achieved for the averaged indices per subject. Averaged very good results using the qualitative scale are in bold*.

Regarding the method of control (Table 2), the main differences appear for the TPR and FP. The fact that MI+Att requires to keep a level of attention makes harder to activate the exoskeleton, but provides less FP. For the same reason, when both paradigms are used, the lags for starting are longer and for stopping shorter, as the time the exoskeleton is going to be moving is shorter. Nevertheless, there is not a significant difference between the %Command of both control methods, because a shorter walking time provides less time walking during walking periods, but less time walking during the last rest period, compensating the %Command value between both events. Therefore, it could be said that MI+Att makes the control safer, but less responsive.

Regarding the subject performance (Table 3), the Acc results vary depending on the subject (*p* < 0.05), with S1 and S2 having values for the classifiers over 75% in average. The subject with lower results is S4, which shows a high TPR 75% with a an Acc around 53.1%, which indicates that the BMI is activating the exoskeleton during the walking events (TPR), but for a short time (low Acc). This irregularity, specifically in the %MI, causes a higher value of FP when the attention paradigm is not considered (see Figure 9). Another fact to take into account is that the last trial uses more information for its model than the second trial, which uses just the first trial. This does not have to be negative in all the cases, since the consideration of an spoiled training trial in the model could be more negative for its use in the classification.

### 3.2. Closed-Loop Results

Once the five training trials were done for each control option, the test trials were registered. They were carried out after the training in sequence. This means five training sessions for MI followed by five tests for MI and then five training sessions for MI+att followed by five tests for MI+att. Table 4 shows the closed-loop results obtained.

**Table 4:**
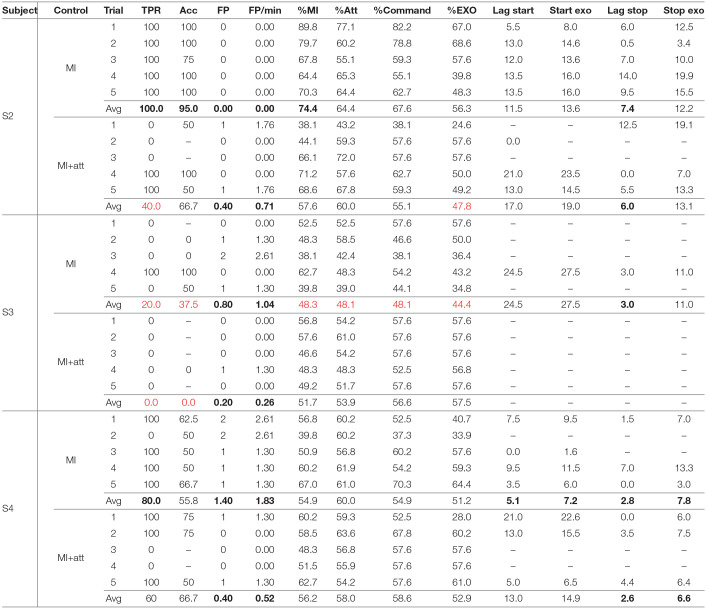
Results of the analysis of the test trials.

*All the tests were done in closed-loop control. Averaged results considered very good using the qualitative scale are in bold, while poor in red*.

Looking at Table 4, some facts can be extracted. The most obvious one is that S3 was not able to make the BMI work in closed-loop control. Rex was hardly activated during the tests. This contrasts with the results obtained by the subject in the opened-loop trials. However, S2 and S4 obtained a good performance for the MI control with a high number of activations of the exoskeleton during walking periods (TPR) and a proper activation of the commands (Acc). Nevertheless, classifier accuracies were a bit lower than in the opened-loop scenario.

In the case of the %EXO index, i.e., the time that the Rex is standing or walking during the correct events, achieved a value of 56.3 and 51.2 for subjects S2 and S4. This, a priori low value, is caused by two causes. First the algorithms need several seconds of correct features in order to achieve a command decision (sections 2.3.2 and 2.3.3). Second, Rex has an inherent lag for responding to the commands which is very variable, especially in the case of a stop, as it depends on the limbs position when the command is issued. This information can be calculated by the difference between Start exo and Lag start, and between Stop exo and Lag stop from the data in Table 4. Rex lags were in average 2.8 *s* for the start and 5.4 *s* for the stop, times which are added to the command lag decision. For instance, looking at Figure 8, which corresponds to the 1st MI test trial of S2, it lags 8 *s* for the start and 12.5 *s* for the stop. This makes a %EXO of only 67.0%, for a excellent classifier trial (89.8%MI and 77.1%Att). Another consequence that can be extracted from the results is that the MI control performs better than the MI+att for S2 and S4. In the same way than in the opened-loop trials, the combination of the paradigms makes the BMI more conservative, avoiding the activation of the Rex in two of the five test trials for both subjects.

## 4. Discussion

The MI paradigm is based on gamma band. This is not a band that it is usually considered in literature and only a few studies prior to our previous research (Ortiz et al., 2019) have considered it (Seeber et al., 2015). Two were the main reasons to focus on this band instead of θ, α, or β bands. First, γ band is less affected by low frequency noise. In the research, an active filter was applied to mitigate eye blinking (H^∞^) and passive mitigation (mesh, clamps) to avoid wire oscillations or muscle noise (no swallowing or chewing). However, as the whole tool works in real time, no other offline mitigation techniques can be applied, such as independent component analysis (ICA) (Delorme and Makeig, 2004), so this band can be less affected by motion noise. Second, γ band is associated with attentive focus (Rao, 2013) and gait attention (Costa et al., 2016; Costa-García et al., 2019). For this reason, the attention level paradigm reinforces the MI paradigm by requiring a high focus of the subject during the walk events. This produces that the proposed BMI obtains sometimes a lower accuracy than other MI paradigms in the literature, but with a lower value in FP/min, which was one of the priority objectives of the research. This can be seen comparing the results of this research with the ones presented in the review of lower-limb exoskeletons by He et al. (2018). FP/min is only provided in the study by Do et al. (2013) achieving a 7.42±2.85 FP/min. This is substantially higher than the FP/min presented in this article, which rarely go beyond 2 FP/min and are in most cases below 1 FP/min. The comparison with accuracy can be hard, as the way it is accounted can vary from different researches. Table 2 of He et al. (2018) varies from 68 to 99% depending on the study. This value can be confronted with the Acc index for the whole BMI or with %MI or %Att for the individual paradigms. Accuracy is in the range of the literature studies except for S4 and the closed-loop trials of S3.

Results by subject also indicate one of the most common problems of BMI studies, which it is the high dependency of the results on the individual. The fact that the tool provides performance indices since the second trial could help to detect subjects that are having troubles with the BMI. This is especially important in the case of ACV or stroke patients, which could also have cognitive difficulties which could make them unable to use the BMI, even if they have been selected as suitable in the previous clinical selection stage. A quick detection of these problems could help clinicians to adapt the therapy in these cases, for instance only applying an opened-loop control to the exoskeleton. In addition, the degree of expertise of the subject with a BMI is a factor that improves the performance. In this study, only one session per subject (except for S2) was carried out, which does not allow to study the evolution of each individual with the different sessions.

Another important factor to consider is the use of erroneous trials to create the model, as it affects the classifier output. One BMI based on attention is more subject to distractions which could cause erroneous training trials, but MI or rest events are also affected by mental distractions. Even as environment conditions can up to a certain point be controlled, mental distractions are hard to detect beyond the subject's feedback. In this research, each training trial is checked in real time with the model created with previous training data which allows to discharge bad training trials to avoid spoiling the posterior performance of the BMI. For instance, the fourth training trial of S4 should have been neglected for model creation due to achieving just a %MI of 28.2% (Table 1). This trial filtering for the model creation was not considered in this research to limit the length of the sessions and to compare all the subjects in the same conditions. However, it will be something to apply in future researches avoiding any trial with %MI or %Att below 70% to improve model output when used in the closed-loop trials.

The proposed BMI has been designed to serve as a tool for rehabilitation therapies helping the subject to keep a high cognitive engagement during a trial. The attention level paradigm helps to improve the FP/min index, but makes the BMI less responsive with lower Acc and TPR, and less activation time of the exoskeleton (%Command). A revision of the command decision rules explained in subsection 2.3.4 could help to improve the results. Another option would be to offer the attention level as a feedback that could enhance the mental engagement of the subject during the walk events, or reduce it during the rest events. Additionally, the order of the application of the control methods could have affected the subjects due to fatigue. A high fatigue produces a low attention to the mental task of MI. Looking at Table 1, this is sustained by the classifier percentages. The training trials value for 7 − 10 trials (MI+att opened-loop trials) were in almost all the cases lower than for 2 − 5 trials which were the ones used for the model of the MI+att closed-loop trials. The length of the experiments is another key factor to consider. As two different methods of control were tested, closed-loop sessions extended to 3 h, indicating the subjects that fatigue was clearly present in the last test trials. This could be the reason of the lower test results of S3 and the MI+att of S2 (Table 4). Protocols must be improved in order to avoid sessions over an hour and a half since the beginning of the preparation of the subject, even if the actual active time of the session is below an hour, all the preparation times must be reduced.

## 5. Conclusions

During this research, a new BMI based on MI in γ band has been tested with a Rex exoskeleton in real time, not only in opened-loop control, but also in closed-loop control. In addition, an innovative BMI to assess the level of attention to gait has been implemented and combined with the former BMI. Two of the experimental subjects were able to control the exoskeleton in closed-loop control with very low FP, which was one of the main objectives to achieve.

Regarding the combination of the attention level with the MI paradigm, it provided similar results in opened-loop trials, but activating the exoskeleton in a more conservative way with slightly fewer FP and times of activation. However, the length of the proposed protocols was so long that the induced fatigue affected the results of the closed-loop test trials. Independently of its use in the closed-loop control, the attention level can be used as a way to give feedback to the subject and to inform the clinical supervisor of the cognitive engagement of the subject.

The experimental sessions fulfilled, show a case of study for the validation of the proposal, which has been validated as a promising technique to operate an exoskeleton in rehabilitation therapies which imply the cognitive engagement of the subject. Future research, will explore how the expertise of the subject can affect both paradigms during several sessions. In addition, the flaws detected in the current proposal will be corrected in future implementations of the BMI, such as limiting the fatigue of the subject with shorter sessions and assuring that the model training trials are not inducing errors in the classifier. All of this, in order to allow its future implementation with non able-bodied subjects in a clinical study.

## Data Availability Statement

The datasets presented in this article are not readily available because of University of Houston IRB restrictions. Requests to access the datasets should be directed to Dr. Contreras-Vidal.

## Ethics Statement

The studies involving human participants were reviewed and approved by Institutional Review Board of the University of Houston, TX (USA). The patients/participants provided their written informed consent to participate in this study.

## Author Contributions

MO and JA: conceptualization of the project. MO: methodology, formal analysis, investigation, and data curation. MO, LF, and EI: software development. MO and EI: validation. JC-V: resources and supervision. MO and LF: writing original draft preparation. MO, LF, EI, JC-V, and JA: writing, review, and editing. MO, JC-V, and JA: project administration and funding acquisition. All authors contributed to the article and approved the submitted version.

## Conflict of Interest

The authors declare that the research was conducted in the absence of any commercial or financial relationships that could be construed as a potential conflict of interest.
